# Longitudinal study of the vaginal microbiome in pregnancies involving preterm labor

**DOI:** 10.20407/fmj.2021-017

**Published:** 2021-11-25

**Authors:** Yoshiko Sakabe, Haruki Nishizawa, Asuka Kato, Yoshiteru Noda, Akiko Ohwaki, Hikari Yoshizawa, Takema Kato, Takao Sekiya, Takuma Fujii, Hiroki Kurahashi

**Affiliations:** 1 Department of Obstetrics and Gynecology, Fujita Health University, School of Medicine, Toyoake, Aichi, Japan; 2 Division of Molecular Genetics, Institute for Comprehensive Medical Science, Fujita Health University, Toyoake, Aichi, Japan

**Keywords:** Vaginal microbiome, Preterm delivery, Lactobacillus iners

## Abstract

**Objectives:**

Alterations in the vaginal bacterial flora reflect the status of various obstetric conditions and are associated with mechanisms that underlie certain pregnancy-associated complications. These changes are also a predictive biomarker for clinical outcomes of these adverse events.

**Methods:**

We examined the vaginal microbiome in samples from pregnant Japanese women with preterm labor.

**Results:**

The microbiota composition in preterm delivery (PD) samples differed from those of control or threatened preterm delivery (TPD) samples in principal component analysis. An increase in Firmicutes and a decrease in Actinobacteria were significantly associated with PD only (both *P*<0.01). In the Firmicutes phylum, *Lactobacillus* tended to be abundant, and the abundance of *L. iners* and *L. crispatus* was especially high, whereas the *L. gasseri* population was low in PD samples. Longitudinal analysis showed that the abundance of *L. iners* decreased after commencing tocolytic treatment in TPD samples compared with before treatment, but it remained high in PD samples.

**Conclusions:**

The vaginal microbiome may be a useful prognostic indicator of preterm labor and a monitoring tool for tocolytic treatment to prevent preterm birth.

## Introduction

Preterm delivery (PD) is the main cause of perinatal mortality and morbidity worldwide.^1^ Preterm infants often suffer from respiratory distress syndrome, necrotizing enterocolitis, and intracranial hemorrhage, which potentially lead to chronic lung disease and developmental delay later in life.^2^ A major challenge in preventing PD is identifying women at the greatest risk. Among the known maternal and fetal genetic factors, as well as various environment factors, up to 50% of all PD cases are associated with microbial etiologies.^3^ The most common pathway to intrauterine infection is ascent from the vagina through the cervix into the uterine cavity. Bacterial vaginosis, which is a condition involving an altered vaginal microbiota, has been consistently identified as a risk factor for PD.^4^ However, the interplay between the microbiota and the host’s physiology remains poorly understood in term and preterm pregnancies. Therefore, to better understand the mechanisms and prognostic biomarkers associated with PD, determining the dynamics of the vaginal microflora is crucial.

Recent advances in metagenomics analysis via high throughput sequencing using next-generation sequencing has enabled evaluation of bacterial diversity among samples under various clinical conditions. Quantitative and qualitative analyses have been conducted using sequence information of 16S ribosomal RNA gene variable regions amplified from vaginal swab samples. Next-generation sequencing-based microbiome studies have provided culture-independent unbiased information on the vaginal microflora. Although a considerable number of studies have been published in this field, inconsistent conclusions have been reached.^5–8^ To further investigate the possible involvement of bacterial infection in the etiology of PD, we examined the vaginal microbiome and evaluated PD-specific bacterial profiles in a Japanese population. We performed not only cross-sectional analysis but also longitudinal analysis, using a series of samples from each woman with preterm labor in our study to minimize the effect of inter-individual variance in the microbiome. We further assessed whether the identified PD-specific bacterial profile had clinical utility for prognostic prediction of preterm labor. Additionally, we screened for key taxa as biomarkers of the response to tocolytic treatment in women with preterm labor.

## Methods

### Subjects

All of the clinical samples were collected at the Department of Obstetrics and Gynecology, Fujita Health University Hospital, Japan. Pregnant Japanese women with preterm labor participated in the study. Preterm labor was diagnosed by the presence of at least two uterine contractions every 10 minutes associated with cervical changes in patients with a gestational age of between 20 and 34 weeks. We classified the births in this cohort as PD (delivered at <37 weeks gestation, n=5) or threatened preterm delivery (TPD) (delivered at term, n=12). Women with an uncomplicated pregnancy who were matched for gestational weeks with those who had PD or TPD were also enrolled as a control group (n=23). The clinical details of these women are shown in Table 1. In women who had PD or TPD, continuous intravenous ritodrine hydrochloride treatment at a dose of 50 μg/min was started and the dose was increased to 75 μg/min according to uterine contractions and the cervical length. The infusion rate was increased to a maximum of 200 μg/min. Informed consent was obtained from each patient and this study was approved by the Ethical Review Board for Clinical Studies at Fujita Health University.

### Sample collection and DNA extraction

Vaginal swab samples were obtained from subjects with PD or TPD at admission and after commencing tocolytic treatment (>30 gestational weeks). Samples were also obtained from women with a normal uncomplicated pregnancy who were matched for the gestational week of the PD and TPD specimens. Briefly, a double-tipped swab (BD BBL CultureSwab plus) was inserted approximately 5 cm into the vagina, pressed against the vaginal sidewall, rotated for 5 seconds, and then removed. Samples were stored at –80°C until later genomic DNA extraction using a QIAamp DNA Microbiome Kit (Qiagen GmbH, Hilden, Germany) in accordance with the manufacturer’s instructions. Seven samples were obtained from 5 women with PD, 20 from 12 women with TPD, and 45 from 23 women with a normal uncomplicated pregnancy.

### Polymerase chain reaction amplification and sequencing of 16S rRNA gene V3–4 regions

Polymerase chain reaction (PCR) reactions to amplify the V3–4 regions of bacterial 16S rRNA were conducted using the following universal primers: forward (5'-**TCGTCGGCAGCGTCAGATGTGTATAAGAGACAG**TCGTCGGCAGCGTCAGATGTGTATAAGAGACAGCCTACGGGNGGCWGCAG-3') and reverse (5'-**GTCTCGTGGGCTCGGAGATGTGTATAAGAGACAG**GTCTCGTGGGCTCGGAGATGTGTATAAGAGACAGGACTACHVGGGTATCTAATCC-3').^9^ The bold letters denote the Illumina overhang adapter. Individual samples were barcoded, pooled to construct the sequencing library, and then sequenced using Illumina Miseq (Illumina, San Diego, CA, USA) to generate pair-ended 2×300 reads. Taxonomies were assigned using GreenGene or Ezbio. For further detailed taxonomies, we used a combination of rdp classifier and SpeciateIT.

### Statistical analysis

For a β-diversity measure, the weighted UniFrac distance matrix, which measures the pairwise difference in microbial diversity among samples, was calculated using QIIME. To provide visualization of the sample distribution patterns, principal component analysis (PCA) was applied to transform the UniFrac distance matrices into principal coordinates. For volcano plotting, fold changes were calculated using QIIME and the statistical significance was calculated using logistic regression analysis. Intergroup comparisons were made using the Mann–Whitney U test or one-way analysis of variance. Because of the exploratory nature of this study, a *P* value <0.05 was considered statistically significant, and *P*>0.05 and <0.1 was recognized as indicating a certain trend toward significance.

## Results

**Figure 1:**
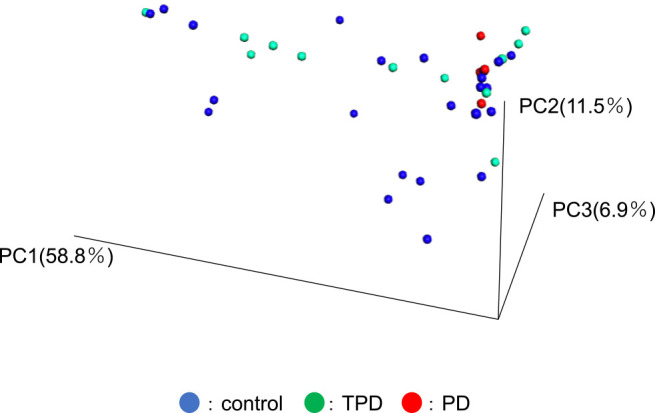
Comparative analysis of vaginal microbiome profiles between the PD and TPD or control groups. A. Alpha diversity of the vaginal microbiome. The rare fraction curve was constructed using weighted UniFrac analysis. Red lines indicate the PD group and blue lines indicate the TPD and control groups. B. A PCA plot was constructed using weighted UniFrac analysis. Red, green, and blue circles indicate the PD, TPD, and control groups, respectively.

We examined the vaginal microbiome of our study subjects by deep sequencing of PCR amplicons of the V3–4 regions in the bacterial 16S rRNA gene. We first conducted PCA analysis for the weighted UniFrac distance matrices to examine whether the global microbiome profile of each sample could differentiate PD from TPD or controls. The PD samples were clustered, while the TPD and control samples were not in clustered this analysis (Figure 1).

**Figure 2:**
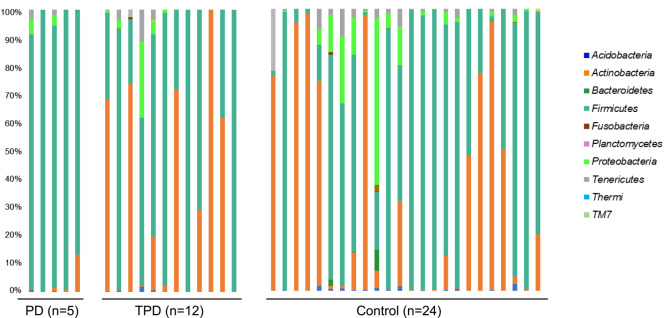
Relative abundance of different bacterial phyla in each sample. The seven most abundant bacterial phyla are indicated. One-way analysis of variance was performed for comparison between each group. Firmicutes: controls vs TPD, *P*=0.84; TPD vs PD, *P*=0.005; controls vs PD, *P*=0.0001. Actinobacteria: controls vs TPD, *P*=0.87; TPD vs PD, *P*=0.01; controls vs PD, *P*=0.001. Proteobacteria: controls vs TPD, *P*=0.61; TPD vs PD, *P*=0.65; controls vs PD, *P*=0.15.

**Figure 3:**
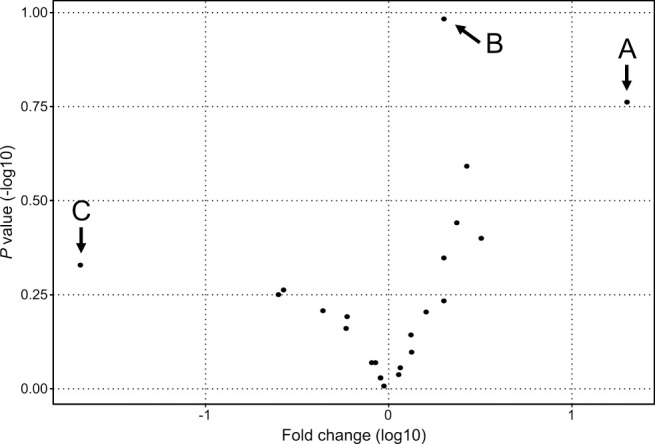
Volcano plot of the relative abundance of each bacterial genus. The x-axis indicates the fold changes of genera in PD samples compared with non PD samples, while the y-axis indicates *P* values. A. *Finegoldia* (20-fold, *P*=0.17); B. *Lactobacillus* (2-fold, *P*=0.10); and C. *Bifidobacterium* (0.02-fold, *P*=0.46).

We next compared the relative abundance of the bacterial populations between the PD group and the TPD or control group via taxonomic assignment. Clear differences were observed when we analyzed the data at the bacterial phyla level. Firmicutes was the dominant phylum in the PD group relative to the TPD or control group (*P*<0.01, PD versus TPD; and *P*<0.01, PD versus controls), while Actinobacteria was dominant in the TPD and control groups (*P*<0.01, PD versus TPD; and *P*<0.01, PD versus controls) (Figure 2). However, when we performed a similar analysis for more detailed bacterial taxa, the differences became statistically unclear. To identify additional bacterial taxa with different abundances between the PD, TPD, and control groups, we performed a similar statistical analysis and expressed the data in a volcano plot. When we analyzed the data for the bacterial genera, three species showed notable trend differences in terms of their abundance between the PD and non PD groups (Figure 3). The sequence reads for *Finegoldia* were the most abundant in the PD group (20-fold, *P*=0.17). *Lactobacillus* showed a lower level than that for *Finegoldia*, but this still tended to be higher in the PD group than in the control group (2-fold, *P*=0.10). Because both of these strains belong to the Firmicutes phylum, we speculated that the increase in *Finegoldia* and *Lactobacillus* in PD may be responsible for the increase of Firmicutes. In contrast, *Bifidobacterium* showed the lowest abundance in the PD group, although this was not significant (0.02-fold, *P*=0.46). We further speculated that the decreased abundance of *Bifidobacterium* in PD underlies the decrease of the Actinobacteria phylum to which it belongs.

**Figure 4:**
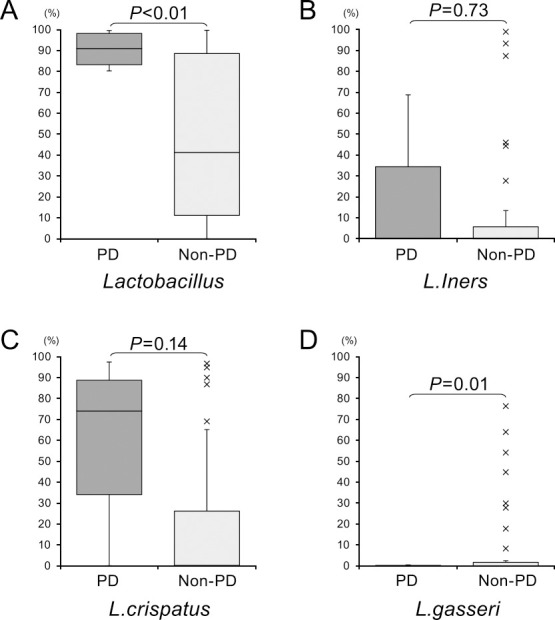
Comparative analysis of the relative abundance of *Lactobacillus* strains in the vaginal microbiome between the PD and non PD groups. A. *Lactobacillus* genus; B. *L. iners*; C. *L. crispatus;* and D. *L. gasseri*.

To specify the bacterial species in the vaginal microbiota that were enriched in PD cases, we performed additional sequence analysis with reference to public databases. We then compared the abundance of the identified strains between the PD and control groups. We did not find any specific *Finegoldia* or *Bifidobacterium* strains that could have contributed to the high or low abundance of these genera, and thus possibly differentiate PD from either TPD or a normal pregnancy. In our analysis of *Lactobacillus* species, we found a higher relative abundance of strains of this genus in the PD group among the bacterial taxa (*P*<0.05). We found that the relative abundance of *L. crispatus* and *L. iners* tended to be higher in the PD group, but this was not significant. In contrast, the relative abundance of *L. gasseri* was significantly lower in the PD group (*P*<0.05; Figure 4).

**Figure 5:**
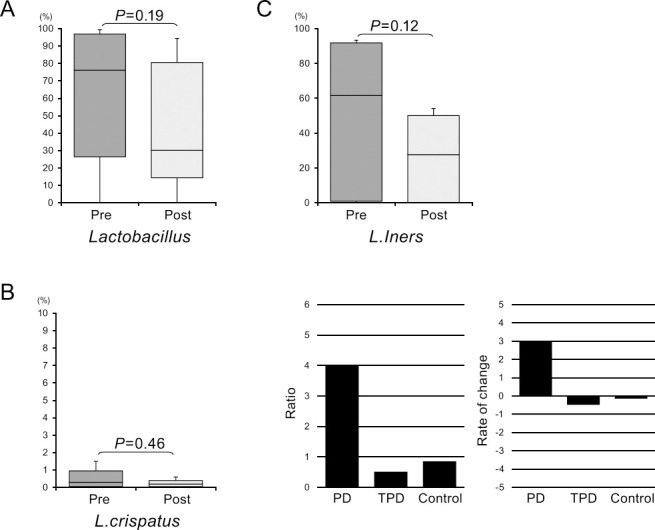
Longitudinal analysis of the abundance of strains of the *Lactobacillus* genus in the vaginal microbiome in the TPD group. The relative abundance of *Lactobacillus* was compared between pre- and post-tocolytic treatment for premature labor. A. *Lactobacillus* genus; B. *L. crispatus*; and C. *L. iners*. We also calculated the post- to pre-treatment ratio for the abundance of *L. iners* in the PD, TPD, and control groups.

Finally, longitudinal analysis was performed using samples obtained from women with TPD who were included in this study during their course of tocolytic treatment. When we compared the abundance of the various bacterial taxa before and after this therapy, the relative abundance of *Lactobacillus* genus strains showed a decline after therapy (Figure 5). Moreover, *L. crispatus* and *L. iners* species were also lower in this treated group. Notably, while the relative abundance of *L. iners* was decreased in the TPD group, it was not reduced in the PD group (Figure 5C) or in the control group. Our findings suggest that the reduced abundance of *L. iners* reflected a response to tocolytic treatment.

## Discussion

The maternal vaginal microbiota contributes to the pathophysiology of preterm labor, but conflicting results in recent years have raised doubts about the validity of this relationship. The microbiome is affected by various factors, such as ethnicity, social and demographic parameters, and the vaginal subsites used for sampling.^10,11^ In our study, to minimalize these variations, we enrolled only Japanese subjects and used similar vaginal subsites for sampling. However, because the microbiome profile varies considerably among individuals, we performed not only cross-sectional analysis, but also longitudinal analysis, which allowed us to better interpret the vaginal microbiome data.

Although PCA showed differences between PD and TPD or control uncomplicated pregnancies, we only found phyla that were specific to PD in our analyses, but not more detailed results for variation in bacterial taxa using standard comparative statistics. Therefore, we used a volcano plot to identify strains of the *Lactobacillus* genus that differed between PD and non PD samples. *Lactobacillus* is the most common type of bacterium in the healthy adult vagina, and its abundance at this site increases during pregnancy and does not change or slightly increases with the progression of gestation.^5,6,12^ Additionally, the abundance level of *Lactobacillus* declines in PD.^6–8,13^ However, this study showed that the vaginal richness of *Lactobacillus* was increased in PD samples. The abundance of *Lactobacillus* in the vagina has been attributed to the requirement for a lower pH caused by these lactic acid-producing bacteria to protect against pathogenic infection. Although we did not measure the vaginal pH in our cohort, the diversity of *Lactobacillus* species may be more important that the overall abundance of the *Lactobacillus* population.^14^ Differences in the prominence of *Lactobacillus* strains in the vaginal microbiome may also be simply due to ethnic differences.^15^

We hypothesize that the balance of different *Lactobacillus* species, but not their overall abundance, is protective of the vaginal environment, and that an imbalance can lead to PD. Our study indicated that the *L. crispatus* and *L. iners* bacterial strains tended to be increased, while the abundance of *L. gasseri* was decreased in PD. These bacterial decreases in PD have also been well documented previously.^16,17^ Additionally, *L. crispatus* contributes to the inhibition of *E. coli* growth, which suggests that this bacterial strain also plays a protective role against PD.^18^ Notably, we found that *L. iners* tended to be increased along with an elevated abundance of *L. crispatus* in PD. Previous studies have shown that *L. iners* is associated with a higher risk of PD.^8,19^ When there is an abundance of *L. iners* because of its low production of lactic acid, the vaginal microflora do not maintain a low enough pH to be protective. This condition allows a higher diversity of pathological species, which leads to a higher risk of PD onset.^20,21^ However, some researchers have reported that the richness of *L. iners* is associated with vaginal cleanliness in terms of pathogens.^22^ Our study showed that the relative abundance of *L. gasseri* was significantly decreased in PD, which is consistent with previous findings of a clear negative association between *L. iners* and *L. gasseri*.^23^ However, one study reported that the abundance of *L. iners* and *L. gasseri* were decreased in PD.^24^ Therefore, the clinical significance of the association of these genera with PD in the vaginal microbiome remains unclear.

Finally, we performed longitudinal analysis of the vaginal microbiome in PD and TPD cases and compared pre- and post-treatment samples. When we calculated the post- to pre-treatment ratios, *L. iners* showed a dynamic change in its abundance. The abundance of *L. iners* is susceptible to change according to the condition of pregnancy.^25^ Therefore, we focused on *L. iners* as a candidate biomarker of PD. One of our investigative approaches was to identify any differences in the abundance of *L. iners* between pre-treatment PD and TPD samples because we speculated that it might serve as a prognostic marker for tocolytic treatment. The pre-treatment abundance of *L. iners* in the PD group was higher than that in the TPD group, but this was not significant. Our second approach was to assess any changes in the abundance of *L. iners* during treatment. The abundance of this bacterium decreased in the TPD group, but not in the PD group, during tocolytic therapy. This finding indicated that the abundance of *L. iners* is potentially a good biomarker of the response to tocolysis.

An important limitation to this study is the variation in the microbiome profile among individuals, which could be overcome by an increased number of samples. Further and more detailed evaluations of the vaginal microbiome using a greater number of sampling sites from each patient and a larger cohort will help to validate its potential usefulness as a biomarker of PD/TPD. Adding living *Lactobacillus* species to correct the vaginal bacterial flora might confer a prophylactic benefit against the risk of PD. However, a recent meta-analysis showed no evidence that taking probiotics or prebiotics during pregnancy decreased the risk of preterm delivery.^26^

In conclusion, the vaginal microbiome may be an effective prognostic indicator of preterm labor and of the effects of tocolytic treatment for preventing preterm birth.

## Figures and Tables

**Table1:** Clinical parameters of the study groups

	PD	TPD	Control	*P* value		
	n=5	n=12	n=23	PD vs TPD	PD vs controls	TPD vs controls
Maternal age (y)	33.0±2.3	32.0±1.8	33.9±0.6	0.74	0.66	0.28
Pre-pregnancy body mass index	21.1±1.3	20.3±0.9	21.3±0.7	0.66	0.87	0.37
Parity	1.8±0.5	1.3±0.3	0.8±0.2	0.41	0.13	0.17
Cervical length (mm)	12.8±3.1	19.3±2.1	39.9±1.5	0.13	<0.01	<0.01
Gestational age at sampling (wk)	27.5±2.7	28.0±1.2	27.8±1.3	0.87	0.92	0.91
Gestational age at delivery (wk)	30.5±2.6	37.8±0.3	38.5±0.3	<0.01	<0.01	0.33
Birth weight (g)	1592.0±516.1	2911.7±158.8	3072.0±72.3	<0.01	<0.01	0.38

Data are mean±standard error.
